# Maternal, Postnatal, and Management-Related Factors Involved in Daily Weight Gain and Survivability of Suckling Zaraibi Goat Kids in Egypt

**DOI:** 10.3390/ani12202785

**Published:** 2022-10-15

**Authors:** Ali Ali El-Raghi, Nesrein M. Hashem

**Affiliations:** 1 Department of Animal Production, Faculty of Agriculture, Damietta University, Damietta 34517, Egypt or; 2 Department of Animal and Fish Production, Faculty of Agriculture (El-Shatby), Alexandria University, Alexandria 21545, Egypt

**Keywords:** goat, risk factors, management, postnatal growth, survival rates

## Abstract

**Simple Summary:**

A pre-weaned Zaraibi goat kid’s body weight gain and survival are complex traits affected by the maternal ability of the doe and the kid’s capability for rapid growth and survival, in addition to the management tactics and environmental variables during the postnatal period. In this study, we investigate the effect of some potential maternal, postnatal, and management-related factors, including sex, litter size, birth weight, daily milk yield, parity, kidding year, and kidding season. The effects of these factors on both the daily body weight (ADG) gain and survivability of Zaraibi (Nubian) kids during the suckling period are studied. The body weight gain for kids was influenced significantly by all the aforementioned factors, except for the kidding season. The framework of the survival analysis revealed that the male kids experienced a greater risk of death compared with the female kids. The single-born kids were at a lower risk of death compared with two or more kids born in a litter. Survival rates increased steadily along with the increase in the mother’s daily milk yield and the dam’s parity number. There was a decrease in the probability of mortality with each passing year. In practice, monitoring traits that indicate an imbalance in milk and that could lower the body weight of the kid could promote the identification of kids with a higher risk of death and the inability to achieve the required body weight gain.

**Abstract:**

During the early period of an animal’s life, rapid growth and higher survival rates can provide more profits to producers by minimizing the rearing and replacement costs. The present study aimed to investigate the effect of some maternal, postnatal, and management-related factors (sex, the litter size [LS], the kid’s birth weight [KBW], the daily milk yield [DMY], and the parity order), as well as the kidding year and season on both the average daily weight gain (ADG) and survivability of suckling Zaraibi goat kids during the postnatal period. A total of 3005 kidding events were available from the herd of Zaraibi goats raised at El-Serw Experimental Station from 2008 to 2014, which belongs to the Animal Production Research Institute. Data revealed that the overall ADG was 131.14 ± 2.34, 94.22 ± 2.21, and 85.63 ± 2.33 g/day, whereas the survival rates were 94.68%, 91.81%, and 90.62% during the periods from birth to 1, 2, and 3 months, respectively. During all periods considered, the aforementioned maternal, postnatal, and management-related factors significantly affected the ADG. In males, the ADG increased compared with females. Singletons had a higher ADG compared with kids born to a litter of two or more. A higher KBW and DMY were associated with an increase in the ADG. Additionally, increasing the parity order was associated with an increased ADG. The kidding season did not have a significant effect on the ADG, whereas the kidding year showed a significant effect on the ADG. During the pre-weaning period, the hazard of death increased by 23.1% (hazard ratio (HR) = 1.231; 95% CI = 0.972–2.057) in males compared with females in terms of survival rates. A higher LS was associated with an increase in the incidence of mortality, increasing by 51.6% (HR = 1.516; 95% CI = 1.363–1.719) with each unit increase in the LS. Additionally, the KBW was an extremely important determinant of survivability, as the risk of death decreased by 62.8% (HR = 0.372; 95% CI = 0.229–0.504) with the increase in the KBW. Likewise, survival rates increased steadily along with an increase in both the DMY and parity number. The risk of death decreased by 52.7% (HR = 0.473; 95% CI = 0.376–0.808) and 38.2% (HR = 0.618; 95% CI = 0.512–1.724) with the increase in the DMY and parity number, respectively. The kidding season did not significantly affect the kid’s survivability, but with each passing year, there was a decrease of 2.8% (HR = 0.972; 95% CI = 0.952–0.991) in the probability of mortality. In conclusion, better growth and survival rates can be achieved by controlling the maternal, postnatal, and management-related factors, as well as upgrading management plans.

## 1. Introduction

Goat farms have the potential to flourish when fostered by the systems of an intensive or semi-intensive nature, which work as a stimulus for commercial goat production. Under the organized management of farms, losses occur because of various causes of death. Thus, some economic productive and reproductive traits have to be improved under these systems to sustain the productivity of the herd [1]. Among these economic traits are the kid’s growth rate and their ability to survive during the postnatal period, determined by the success of the production process in raising kids. These are the main sources of culled animal replacement and meat and milk production [2]. Improving these economic traits may contribute to deflating the gap between the demand associated with meat production and the ability to produce enough to meet that demand, ensuring production at a global scale and food security.

Zaraibi goats are considered to be one of the well-known native Egyptian breeds, also called Egyptian Nubian goats. This breed is widely kept in Egypt and the Near East region because of their distinguished productive and reproductive traits, such as being highly prolific and producing sufficient amounts of high-quality milk and meat. The average body weight of a Zaraibi goat is 25–50 kg. It produces 150–300 kg of milk each season, and its rate of producing twins is approximately 2.5%. Therefore, the Zaraibi breed of goats has become a target for a genetic improvement scheme. Early growth is an important factor in gaining profits via meat production [3]. Former studies have demonstrated the existence of a discrepancy in the rates of growth and survival during the postnatal period of suckling kids because of the presence of several explanatory variables that mainly affect these economically positive traits [4,5].

Published studies on Zaraibi kids under Egyptian management conditions are scarce, although several studies have identified the factors related to the performance of animal growth and the survivability of suckling kids and lambs. To address this data gap, the present study was devoted to exploring the potential effect of some maternal, postnatal, and management-related factors including sex, the litter size (LS), the birth weight (BW), the daily milk yield (DMY), and the parity number (PA), as well as the kidding season (SE) and the year (Y), on the growth rates and survivability of Zaraibi kids during the pre-weaning period. Moreover, to the best of our knowledge, this is the first study that used the product-limit method of Kaplan and Meier (survival analysis) to estimate the survival rates of Zaraibi suckling goat kids under Egyptian conditions. Usually, the limitation in the analysis of such a variable is related to data availability through the time for each individual. Time progress is the main basis for survival analysis, that is characterized by a certain relationship between the two events through the time (e.g., a kid’s birth and death). For this reason, survival analysis is adopted to tackle the lack of data related to a certain event. The survival analysis can efficiently tackle the issue of censoring as its main variable, other than time, and it also addresses whether the expected event happened or not. This advantage is important for studying events such as a mortality rate in livestock farms, which is time dependent. Applying survival analysis allows for the use of records from surviving (censored) and dead (uncensored) animals/kids, as both records can be used jointly and introduced into the analysis, therefore all the available data about the animals/kids are properly used [6,7]. The obtained data might permit managers to follow better management techniques to reduce mortality rates and maximize profits.

## 2. Materials and Methods

### 2.1. Animal Management and Feeding

The goats were kept in open barns with shelters and received nutritional requirements that fulfilled their needs, as recommended by the National Research Council, at all productive stages [8]. According to the feeding system applied at most animal farms in Egypt, green Egyptian clover (*Trifolium alexandrinum*) was offered in winter and spring, whereas clover hay was provided in summer and fall, with crop stubble or rice straw as an addition or a substitute. Fresh water and mineral blocks were consistently offered. The nulliparous doe begins mating approximately at 15 months of age with an average body weight of 30–35 kg. A flushing period of 2 weeks premating was applied, during which a 0.25 kg/doe/day concentrate supplement was offered. From pre-parturition to the first week of lactation, the concentrate supplement was offered again for 2–4 weeks. Two breeding seasons were launched every year where half the number of the does were introduced to bucks in autumn and had their kids in the spring and the other half were introduced to bucks in the spring and had kids in autumn. Random selections were conducted to split the does into groups during each breeding season. A group of 25–30 does were given to each fertile buck. To prevent inbreeding, each buck was allowed to mate with the same does for only two seasons. After those two seasons, another buck from the same flock was substituted. Once the kids were born, the newborns instantly received nutrition in the form of the mother’s secreted colostrum, and iodine dye was applied to the wound that remained after the umbilical cord was removed. To establish the mother–kid bond, they were not separated from each other for 3 months postpartum, and the next step was to transfer the dams and young kids to individual pens for 3 to 4 days. Identification numbers were given to the newborn kids who did not die within 2 to 4 h after birth. Cases other than these were categorized as either a stillbirth or neonatal death. Following the complete drying of the body, the live BW of the newborn kids was recorded within a maximum of 6 h after the birth and then at monthly intervals by using a digital balance. The individual milk yield was determined on a weekly basis by keeping the newborn kids in separate pens for 20 h and milking the goats by hand until the udder was empty throughout the suckling period. The milk yield of each goat was recorded and multiplied with a correction factor of 1.2 to obtain the DMY [9].

### 2.2. Data

All survival data used for the present study were based on recordings from a Zaraibi herd raised at the El-Serw Experimental Station belonging to the Animal Production Research Institute (APRI), Ministry of Agriculture, Giza, Egypt. From 2008 to 2014, a total of 3005 kidding events were collected. The records of the studied traits, including the animal identity number, date of birth, date of death, sex, litter size, kidding season, birth year, birth weight, monthly body weights, and dam’s parity number, were obtained from the APRI recording system. All incomplete and/or irregular records were removed from the analysis. Calculating the period that lasted from the date of birth until the date of death gave us the failure time. A record of the animals categorized as “right censored” was made, which included the ones that managed to survive until the time of weaning or changed stations by that time. Additionally, the time of exit lasted for 90 days, which was the same for all of the goats (the standard age for weaning at the experimental station).

### 2.3. Statistical Analysis and Model Procedure

The data were edited in MS Excel (Microsoft Corporation, Redmond, WA, USA). Before conducting the main statistical analysis, the data were checked for normality. The mean average daily weight gains (ADG; g/kid) were expressed as ADG = [(FBW − IBW)/duration], where FBW is the body weight at the end of each month and IBW is the initial body weight or BW. A MIXED procedure (PROC MIXED; [10]) was used to analyze the fixed factors affecting the ADG including the sex, LS, KBW, DMY, SE, parity order, and kidding year, whereas the animal ID was introduced as a random factor.

The statistical model was incorporated as the following:

(1) $${\;Y}_{\mathrm{ijklmnop}}=µ+{\mathrm{Ani}}_{i}+S_{j}+{\mathrm{TLS}}_{k}+{\mathrm{KBW}}_{l}+{\;\mathrm{DMY}}_{m}+{\;\mathrm{SE}}_{n}+{\;\mathrm{PA}}_{o}+{\;Y}_{p}+e_{\mathrm{ijklmnop}}$$

where Y_ijklmnop_ is the ADG, μ is the overall mean, Ani_i_ is the effect of ith animal ID, S_j_ is the effect of jth sex (two levels: male and female), LS_k_ is the effect of kth litter size (three levels: single, twins, and triplets or more), KBW_l_ is the effect of lth birth weight of a kid (three levels: 1, 1.5, and ≥2 kg), DMY_m_ is the effect of mth DMY (three levels: <800, 800–1000, and >1000 g), SE_n_ is the effect of the nth kidding season (two levels: spring and autumn), PA_o_ is the effect of the oth parity (five levels: 1st, 2nd, 3rd, 4th, and ≥5th), Y_p_ is the effect of the kidding year (2008–2014), and e_ijklmnop_ is the random error related to each observation. The differences between the means were detected according to Duncan’s Multiple Range Test [11]. A multiple linear regression equation was used to assess the relative importance of the maternal, postnatal, and management-related factors to the ADG according to the REG procedure of the statistical analysis system [10].The product-limit method of Kaplan and Meier [12] was used to estimate the survival of the kids with a consideration of the censored data:

(2) $$S_{t}=\underset{t_{i}\leq t}{\prod }1-\frac{di}{ni}$$

where t_i_ is the periods considered at any point i, d_i_ is mortality till point i, and n_i_ is the number of kids exposed to risk just prior to t_i_. The Cox proportional hazard model was used in the study through PROC PHREG of the statistical analysis system to study the effects of the potential risk factors on survival rates [10]. The Cox proportional hazards regression model is as follows:

(3) λi (t)=λ0(t) exp (β1Xi1+ β2Xi2+···+ βp Xip)

The previous equation can be fitted as a linear fixed-effects proportional hazard model by taking natural logarithms as follows:

(4) In [λi (t )]=In[λ0(t )] (β1Xi1+ β2Xi2+···+ βp Xip)

where λi (t) is the hazard function and λ0 (t) is the baseline hazard corresponding to the probability of dying when all the explanatory variables are zero. T is the timescale of choice. The ratio of h (t)/h0 (t) is the hazard for the failing individual that provides an estimate of the risk per unit change in the explanatory variables relative to the baseline hazard function [13]. Β1, β2 … βp are regression coefficients for different covariates specified in the model, X_i1_ is the covariate value for covariate 1 for individual i, etc. The mortality rates were calculated as the number of dead kids divided by the total number of kids born, whereas survival rates were calculated as the number of live kids (censored) divided by the total number of kids born. Statistical significance was set at a *p*-value of <0.05.

## 3. Results

### 3.1. Maternal, Postnatal, and Management-Related Factors Involved in ADG

The sex, LS, KBW, DMY, SE, PA, and Y were studied to ascertain their effects on the ADG of Zaraibi suckling kids (Table 1). The ADG was 131.14 g during the period from birth to 1 month and decreased by 37 g and 46 g/day during the periods from birth to 2 and 3 months, respectively. Compared with female kids, males had a significantly higher ADG during all periods considered. Additionally, another factor that significantly affected the ADG during all periods considered was the LS. This was maximized for both single- and twin-born kids (*p* < 0.05) compared with triplets or more. It is noteworthy that the higher the KBW, the higher the ADG during the suckling periods, and the differences in the ADG per day between suckling kids with an average KBW of 1.5 and 2 kg were not significant (*p* > 0.05) during the first month of life, whereas a significant difference (*p* < 0.05) was observed during the second and third months that was in favor of the kids with an initial body weight of 2 kg. Additionally, the daily milk yield had a significant effect on the ADG of suckling kids during the postnatal periods until the second month, and the non-significant differences (*p* > 0.05) were shown between kids born to does with the DMY in the category of 800–1000 g, and those with a milk yield more than 1000 g. Does in their fourth and fifth parities had suckling kids with an ADG significantly higher during all suckling stages than kids who were born to primiparous does. The Y significantly affected the ADG over all suckling periods with a significant increase being observed during the latest years. However, the SE did not have a significant effect on the ADG at any period from birth to weaning.

### 3.2. Relative Importance of the Maternal, Postnatal, and Management-Related Factors to ADG

The relative importance of the maternal, postnatal, and management-related factors is presented in Table 2. During the early period from birth to 1 month, the magnitude of the t-statistics indicated that S is the most significant independent variable, followed by the LS, KBW, DMY, Y, and then PA, while SE occupied the latest place in the variables order. The aforementioned order of the relative importance of such independent variables changed during the period from birth to 2 months to be the S, KBW, DMY, LS, PA, Y, and SE. Meanwhile, they were the LS, S, KBW, PA, Y, SE, and DMY during the whole suckling period from birth to 3 months.

### 3.3. Maternal, Postnatal, and Management-Related Factors Involved in Survival Rate

**Figure 1:**
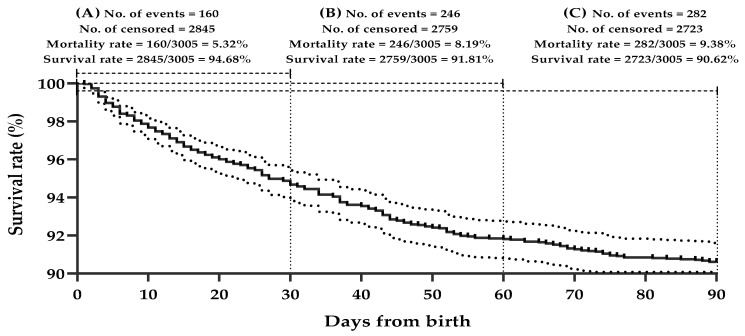
Estimated survival times of Zaraibi suckling goat kids during the periods from birth to 1 month (**A**), birth to 2 months (**B**), and birth to 3 months (**C**), the censored kids were observed on the line marking; however, the outer dotted lines expressed a confidence interval (95%) for the estimated survival function according to the Kaplan–Meier method.

The survival functions related to the three suckling periods according to the Kaplan–Meier method are presented in Table 3 and Figure 1. Interestingly, the survival curves estimate the probability of mortality occurring on a specific day. The overall survival rate was 90.62% during the pre-weaning period; however, the mortality rates were found to be 5.32, 2.87, and 1.19% during the first, second, and third months of age, respectively.

Table 4 provides the results of the tested proportional hazard assumptions. For the LS, the log hazard ratio (HR) was positive, which is interpreted as 29.8% (HR = 1.298) to increase the probability of the death of kids for each unit increase in the LS during the period from birth to 1 month after adjustment has been made for the other variables in the model. The previous proportion increased to 49% (HR = 1.490) and 51.6% (HR = 1.516) during the periods from birth to 2 and 3 months, respectively. By contrast, the log hazard (b) was negative for the KBW, meaning that there was a reduction in the probability of mortality with the increasing KBW being 77.2% (HR = 0.228), 67.6% (HR = 0.324), and 62.8% (HR = 0.372) during the periods from birth to 1, 2, and 3 months, respectively. The amount of suckled milk increased along with a decrease in the predicted probability of a dead kid. The effect of the DMY on the survival rate of kids began to decrease with age during the periods from birth to 1, 2, and 3 months. The risk of death decreased by 80.1% (HR = 0.199), 56.7% (HR = 0.433), and 52.6% (HR = 0.473), respectively. Sex did not affect the hazard rate during the periods from birth to 1 or 2 months (*p* = 0.9994 and 0.4175, respectively), but it had a tendency to affect (*p* = 0.0843) the kids during the third month. Collectively, the probability of male death increased by 23.1% (HR = 1.231) during the whole suckling period compared with females. Similarly, PA did not have significant effects (*p* = 0.1524) on the hazard of death in kids during the period from birth to 1 month, whereas it tended to affect the hazard of death during the periods from birth to 2 and 3 months (*p* = 0.0851 and 0.0517, respectively). The probability of the occurrence of mortality decreased by 29.8% (HR = 0.702) and 38.2% (HR = 0.618), respectively. The Y significantly affected the hazard rate during all studied periods. There was a decrease in the probability of mortality in kids with each passing year by decreasing factors of 0.976, 0.975, and 0.972 during the periods from birth to the first, second, and third months, respectively. The SE had an insignificant effect on the mortality rate of kids during all postnatal periods until weaning, contrary to the results observed for the kidding year.

## 4. Discussion

### 4.1. Factors Involved in ADG and Their Relative Importance

The two most important economic traits that concern goat holders are the growth performance and survivability of suckling kids [2,14]. Growth performance is regarded as a vital indicator of animal well-being, productivity, and profitability [2]. An important element of sustainable productivity is selective breeding, which depends on body weight gain to a great extent as a criterion for the selection process used to improve production [15].

The studied maternal and prenatal factors including the S, LS, KBW, DMY, and PA had significant effects on the ADG of suckling kids according to the present results. Male kids outgrew the females throughout all of the different life stages [15,16]. The superiority of the growth rate of males may be attributed to the anabolic roles of androgens, which contribute to the growing process. It may also be because of their higher consumption of feed and milk resulting from their higher level of activity [17]. In agreement with the findings of former studies on different breeds, the present results showed that the ADG of male kids was superior compared with that of females during all suckling periods [2,4]. The litter size and KBW are considered to be other factors that affect the ADG during the pre-weaning period, as single-born kids had a higher ADG on average compared with multiple-born kids [18]. Additionally, a positive correlation was observed between the KBW and ADG during the aforementioned period [19,20]. This demonstrates the superiority of single-born kids in terms of their BW; thus, the pre-weaning growth rate may be attributed to the prenatal competition between fetuses for nutrients in the uterus [4]. The other probable reason for the superiority of single-born kids during the postnatal period is the lack of competition for milk. In the case of multiple births, does have lower cotyledons than their counterparts with singletons, and there is a significantly positive correlation (r = 0.64) between the BW and the weight of cotyledons [21]. In the present study, the ADG of triplets was significantly lower than single-born kids and twins during all suckling phases. Moreover, the ADG increased steadily with an increased BW.

During the period from birth to weaning, the growth of neonates mainly depends on the milk yield of their dams. Therefore, the offspring’s growth rate is directly influenced by both the quantity and quality of suckled milk during this period [22]. In the present study, the growth rates of suckling kids greatly increased as the amount of suckled milk increased. The present results corresponded with Bogdanović et al. [23] finding that the growth of kids up to 90 days of age is greatly influenced by their mother’s milk yield. Conversely, Žujović et al. [22] reported that the ADG of Balkan suckling kids increased (*p* < 0.001) from the first lactation onwards. In this study, the highest values were for those born to mothers in later lactation seasons (124 g milk/day), whereas the lowest growth rate was observed for kids born to the youngest does (106.5 g milk/day).

Several studies have shown the significant effects of PA on the early growth performance of kids from different goat breeds (Abergelle goats [24] and Ethiopian goats [25]). A significant association between the parity order and the ADG was revealed in the present study. The more dams that were advanced in the parity number, the more they produce offspring with a high ADG during the pre-weaning period. This could be explained by the fact that the milk production and pelvic size increased as the longevity of the dam increased [26,27]. The significant increase in the average growth rate per kidding year may be attributed to the promotions in the management and husbandry systems from one year to another in the present study.

Collectively, the present results indicated that the S, LS, KBW, and DMY were the major four factors responsible for the total variation of the ADG, followed by Y and PA, during the early period of an animal’s life. The present results corresponded with Žujović et al. [22] who showed a significant regression coefficient for BWG from birth to 30 days on the PA and DMY of domestic Balkan goat kids. Bushara et al. [28] reported a positive relationship between the average daily gain and both the LS and S in suckling Taggar goat Kids. Similarly, Yıldırı et al. [29] demonstrated a significant positive relationship between the live body weight at birth and the ADG in Karayaka male lambs.

### 4.2. Factors Involved in Survival Rate

During the suckling period, the higher mortality of kids is one of the major factors that have a negative effect on farm profits. This causes a decrease in the number of animals available for replacement, as well as a decrease in the number of animals that reach a marketable age. There are several numbers of immunological, physiological, and behavioral challenges affecting the survival rates of pre-weaned animals [30]. In well-managed flocks, a 3% rate of neonatal lamb mortality with an upper limit of 5% has been suggested as an acceptable mortality rate for neonates [31]. The estimate of neonatal kid mortality during the first month (5.12%) in the present study was marginally above the upper limit. Remarkably, the overall pre-weaning mortality rate was 9.38% in the present study. Upadhyay et al. [1] found a similar trend in which the pre-weaning mortality rate was 10.93%. These data collectively highlight the fact that the standard farming/management conditions are still inadequately known by many farmers, and more efforts are required to achieve the minimal mortality rate of neonates during the pre-weaning period.

In this study, the majority of mortality in kids occurred during the first month of age (160/282, 56.74%), followed by the second (86/282, 30.49%) and third months (36/282, 12.77%). Similar results were observed in previous studies conducted on kids and lambs, where approximately 69% of all the pre-weaning mortality of kids [32] and approximately 50% of all the pre-weaning mortality of lambs [19,33] occurred during the first month of age. In this study, the data analysis showed that a higher KBW was significantly associated with a lower occurrence of postnatal mortality. Similar results were obtained in other studies. Chauhan et al. [30] reported that the risk of death in Sirohi kids decreased by 78% (HR = 0.22; 95%, CI = 0.18–0.26) with an increased BW. In Deccani sheep, better survival rates were observed with higher BWs, and the risk of death decreased by 47% (HR = 0.530; 95%, CI = 0.40–0.69) with each unit increase in the BW [34]. The ability of heavier kids to maintain their body temperature due to the increased brown fat stores in their bodies is the result of the positive effect of the KBW. Thus, they can start suckling within a very short period after birth, whereas starvation or death due to hypothermia usually awaits kids with a less than average weight [35].

The present study showed that the LS was associated with a higher risk of death with each unit increase with respect to parity. The lower mortality rate in singletons compared with that in twins and triplets may be attributed to the absence of aggressive behavior and competition between animals for suckled milk [36,37]. By contrast, Bangar et al. [34] reported a lower risk of death in twins of 21% (95% CI = 0.32–1.96).

Regarding the effects of parity order, the results of this study showed that the risk of death in suckling kids tended to decrease (*p* = 0.051) by 38.2% during the whole suckling period with an increase in the parity numbers. These data are in agreement with those obtained by El-Abid and Abu Nikhaila [14], who reported that the kids born to does at the first parity showed lower survival rates compared with their counterparts born to older does. The mortality rates were 50.0%, 22.22%, and 27.78% for first, second, and third parties, respectively. In the same context, Al-najjar et al. [37] found that the parity order of Shami goats affected the survival of kids at birth and during the suckling period because of the age of the does, as the survival rate for the kids improved with the increased maturity of their dams. These findings were explained by an incomplete physical structure in doe at early ages and its association with the occurrence of the mortality rate of kids after birth due to having suboptimal production of both colostrum and milk, affecting the immunity or starvation of kids. Other reasons for a higher mortality rate during the first parity may be attributed to the maternal behavior of does and their low ability to recognize neonates and develop a mother–neonate bond.

Milk production is considered to play an important part in the mothering ability of a doe and is mainly influenced by the availability of natural feed resources, breed, litter size, birth season, and doe maturity [38,39]. Providing enough feed for dairy goats before and after parturition would give better results for the DMY [40]. The mortality rates of kids are negatively correlated with the increased milk production of their dams [39]. Suckling is the natural mean by which colostral immunoglobulins are transferred to neonates. Therefore, it is generally recommended to allow a kid to suckle its mother for the first 2 days postpartum to increase their rate of survival [41]. The major effect of the milk yield on the survival rate of kids was observed during a younger age. The present results coincide with Miah et al. [39], who found a linear trend between the survival rates and milk production as the survival rates increased from 47.5% to 70.5% when milk production increased from 80–200 to 400–600 g/day.

In the present study, the risk of mortality tended (*p* = 0.084) to increase by 23.1% in male kids compared with that in females during the whole suckling period. Similar results were found for Harnali lamb using survival analysis for estimating lamb survival up to weaning [42]. Sawalha et al. [36] reported that the risk of death in male lambs is 54% higher than in females (HR = 1.54 ± 0.18). Similarly, Bangar et al. [34] found that male lambs are at a higher risk of death (37%) compared with females (HR = 1.37; 95% CI = 1.05–1.80). Moreover, in Holstein–Friesian cows, Mee et al. (2008) reported that the odds of perinatal mortality in male calves were 1.12 times (95% CI = 1.06–1.17) greater than they were in females. By contrast, Barazandeh et al. [43] did not report significant differences in survival rates between male and female Kermani lambs in Iran. Male kids and lambs differ from their female counterparts as the males have a lesser ability to stand, seek the udder, suckle, or perform other survival-based behaviors compared with females [32]. Moreover, several studies showed that the risk of malpresentation increases with male lambs, and the delivery procedure takes a longer time [44]. Thus, male newborns have a higher probability of suffering from starvation [45].

Regarding the kidding year and season, our data revealed that with each passing year, there has been a decrease in the probability of 2.4, 2.5, and 2.8% for death in kids during the periods from birth to 30, 60, and 90 days, respectively. These results could be explained by the overall improvement in the administration systems in the later years. Our results agreed with the findings of Chauhan et al. [30], who reported that during the whole suckling period, the probability of death (HR = 0.972; 95% CI = 0.952–0.991) in Sirohi kids decreased by 2.8% with each passing year. Finally, the kidding season did not affect the mortality rates of kids according to our results. This may be because of the preventive administrative tactics followed by the farmers in terms of breeding seasons, as two breeding seasons were carried out each year to allow does to give birth in both the spring and autumn when the temperature range suits the thermo-neutral zone of animals (no severe heat or cold)

## 5. Conclusions

The information generated from the multivariate analysis and Cox regression model in the present study showed that sex, birth weight, litter size, parity, and milk yield were the most important risk factors for the average daily weight gain and survival rate of suckling Zaraibi kids during the pre-weaning period. The control of these factors early at the first month of a kids’ age might minimize later losses in goat kids related to productive wastes either due to a low average daily weight gain or due to the survivability of neonates, as the highest average daily weight gain and mortality rates were observed at this age. There is a scope for increasing the birth weight and milk yield, e.g., through the improved nutrition of the dam. Moreover, special attention and specific management practices should be given for females at their earlier parties and multi-born kids.

## Figures and Tables

**Table 1:** Average daily weight gain (ADG) in different categories of fixed factors during different suckling periods (LSM ± SE).

Factors	Suckling Periods					
	*n*	ADG1 (g/Day)	*n*	ADG2 (g/Day)	*n*	ADG3 (g/Day)
Overall means	2845	131.14 ± 2.34	2759	94.22 ± 2.21	2723	85.63 ± 2.33
S						
Males	1462	144.27 ± 1.71 ^a^	1418	104.52 ± 1.18 ^a^	1396	98.15 ± 2.21 ^a^
Females	1383	120.81 ± 2.38 ^b^	1341	86.63 ± 2.09 ^b^	1327	76.12 ± 1.95 ^b^
*p*-value		<0.001		<0.01		<0.01
LS						
1	151	147.42 ± 4.23 ^a^	115	103.52 ± 3.31 ^a^	99	100.21 ± 4.52 ^a^
2	1641	139.10 ± 2.16 ^a^	1611	98.22 ± 1.54 ^a,b^	1601	91.32 ± 3.60 ^a^
≥3	1053	110.27 ± 2.44 ^b^	1033	87.15 ± 1.72 ^b^	1023	71.36 ± 2.20 ^b^
*p*-value		<0.001		<0.01		<0.001
KBW, kg						
1	77	102.56 ± 5.11 ^b^	45	91.36 ± 5.17 ^b^	28	77.96 ± 3.62 ^b^
1.5	1648	144.75 ± 1.79 ^a^	1614	98.21 ± 1.36 ^a,b^	1604	82.86 ± 1.85 ^b^
≥2	1120	152.32 ± 2.08 ^a^	1100	110.50 ± 2.10 ^a^	1091	104.23 ± 2.74 ^a^
*p*-value		<0.001		<0.01		<0.01
DMY, g						
<800	956	104.63 ± 3.37 ^b^	925	81.74 ± 2.63 ^b^	906	78.63 ± 1.50
800–1000	1375	144.52 ± 2.21 ^a^	1340	101.63 ± 1.49 ^a^	1329	82.32 ± 1.39
>1000	514	151.77 ± 4.11 ^a^	494	107.42 ± 2.68 ^a^	488	87.02 ± 4.11
*p*-value		<0.001		<0.01		0.4521
SE						
Spring	1377	132.71 ± 3.01	1324	97.62 ± 2.02	1308	85.22 ± 0.001
Autumn	1468	129.42 ± 1.97	1435	93.41 ± 1.26	1415	89.69 ± 0.001
*p*-value		0.8561		0.2945		0.4410
PA						
1st	715	117.22 ± 1.72 ^b^	690	81.21 ± 1.52 ^b^	676	73.20 ± 1.15 ^b^
2nd	633	126.85 ± 2.33 ^b^	620	92.42 ± 2.30 ^a,b^	611	76.08 ± 2.29 ^b^
3rd	562	139.62 ± 2.69 ^a^	544	93.63 ± 2.63 ^a,b^	538	91.18 ± 2.38 ^a^
4th	449	145.82 ± 4.41 ^a^	432	103.43 ± 4.12 ^a^	428	96.89 ± 3.85 ^a^
≥5th	486	144.08 ± 4.82 ^a^	473	104.85 ± 5.12 ^a^	470	97.33 ± 4.44 ^a^
*p*-value		<0.01		<0.01		<0.01
Y						
2008	452	128.42 ± 2.17 ^b^	437	97.41 ± 1.01 ^a,b^	434	85.32 ± 1.12 ^a,b,c^
2009	486	108.63 ± 1.07 ^c^	471	81.79 ± 2.24 ^b^	464	73.22 ± 1.98 ^c^
2010	417	121.92 ± 3.11 ^b,c^	404	83.33 ± 1.89 ^b^	397	77.87 ± 2.17 ^b,c^
2011	463	123.17 ± 1.42 ^b,c^	451	94.88 ± 1.98 ^a,b^	445	81.69 ± 1.63 ^b^
2012	460	139.84 ± 2.78 ^a,b^	449	98.95 ± 2.18 ^a^	444	87.71 ± 1.17 ^a,b^
2013	293	144.13 ± 4.93 ^a^	282	102.24 ± 3.58 ^a^	277	95.10 ± 1.45 ^a^
2014	274	145.59 ± 4.87 ^a^	265	103.11 ± 3.13 ^a^	262	97.27 ± 2.11 ^a^
*p*-value		<0.001		<0.01		<0.01

*n*, the number of records; ADG1, daily weight gain from birth to 1 month; ADG2, daily weight gain from birth to 2 months; ADG3, daily weight gain from birth to 3 months; S, sex; LS, litter size; KBW, kids birth weight; DMY, daily milk yield; SE, kidding season; PA, parity order; and Y, kidding year. ^a–c^ means with different superscripts in the same column within each variable are significantly different (*p* < 0.05).

**Table 2:** Multiple linear regression analysis of ADG on maternal, postnatal, and management-related factors during suckling periods of Zaraibi goat kids.

Suckling Periods	Factors	b-Reg	SE	T-Value	*p*-Value	Rank
**ADG1, g/day**	S	−24.201	1.128	−21.46	<0.001	1
	LS	−18.015	1.817	−9.91	<0.001	2
	KBW, kg	50.060	5.564	9.00	<0.001	3
	DMY, g	0.233	0.027	8.54	<0.001	4
	SE	−1.400	0.916	−1.06	0.285	7
	PA	7.300	1.482	2.94	0.012	6
	Y	5.035	1.710	4.93	<0.001	5
**ADG2, g/day**	S	−16.666	1.054	−15.81	0.001	1
	LS	−7.500	1.077	−6.96	0.002	4
	KBW, kg	18.660	1.364	13.68	0.001	2
	DMY, g	0.125	0.016	7.44	0.001	3
	SE	−2.861	1.174	−2.68	0.174	7
	PA	5.620	0.969	5.80	0.010	5
	Y	2.833	0.067	4.22	0.005	6
**ADG3, g/day**	S	−22.331	1.054	−11.05	0.001	2
	LS	−15.170	1.372	−21.19	<0.001	1
	KBW, kg	27.033	4.101	6.59	0.003	3
	DMY, g	0.0100	0.005	1.73	0.126	7
	SE	−2.011	0.816	2.45	0.130	6
	PA	6.800	1.301	5.23	0.003	4
	Y	3.214	1.142	2.81	0.007	5

b-reg, regression coefficient; SE, standard error; ADG1, daily weight gain from birth to 1 month; ADG2, daily weight gain from birth to 2 months; ADG3, daily weight gain from birth to 3 months S, sex; LS, litter size; KBW, kids birth weight; DMY, daily milk yield; SE, kidding season; PA, parity order; and Y, kidding year.

**Table 3:** Cumulative survival and mortality rates of Zaraibi goat kids during the different suckling periods from birth to 1, 2, and 3 months.

Items	Suckling Periods		
	Birth to 1 Month	Birth to 2 Months	Birth to 3 Months
No. of events	160	246	282
No. of censored	2845	2759	2723
Mortality rate (%)	5.32	8.19	9.38
Survival rate (%)	94.68	91.81	90.62

**Table 4:** Analysis of maximum likelihood estimates for different potential risk factors affecting the survivability of Zaraibi kids during suckling periods.

Suckling Periods	Factors	b	SE	Chi-Square	*p*-Value	Hazard Ratio	95%, CI
	S (female; ref.)	−0.0001	0.159	0.000	0.9994	1.000	0.732–3.367
**Birth to 1 month**	LS	0.260	0.122	4.500	0.0339	1.298	1.020–1.652
	KBW, kg	−1.479	0.341	18.765	<0.0001	0.228	0.117–0.445
	DMY, g	−1.614	0.263	12.632	0.0001	0.199	0.158–2.087
	SE (spring; ref.)	−0.033	0.120	0.076	0.7825	0.967	0.764–2.224
	PA	−0.121	0.017	1.635	0.1524	0.885	0.695–1.725
	Y	−0.023	0.007	8.010	0.0492	0.976	0.946–1.028
	S (female; ref.)	0.103	0.128	0.657	0.4175	1.109	0.863–2.426
**Birth to 2 months**	LS	0.398	0.010	16.212	<0.0001	1.490	1.227–1.610
	KBW, kg	−1.128	0.276	16.692	<0.0001	0.324	0.188–0.556
	DMY, g	−0.835	0.251	9.526	0.0015	0.433	0.361–0.601
	SE (spring; ref.)	−0.047	0.158	0.091	0.7627	0.953	0.699–2.301
	PA	−0.354	0.177	2.637	0.0851	0.702	0.515–1.145
	Y	−0.024	0.009	8.363	0.0422	0.975	0.948–1.004
**Birth to 3 months**	S (female; ref.)	0.207	0.120	2.980	0.0843	1.231	0.972–2.057
	LS	0.416	0.092	20.023	<0.0001	1.516	1.363–1.719
	KBW, kg	−0.988	0.246	16.016	<0.0001	0.372	0.229–0.504
	DMY, g	−0.747	0.263	7.695	0.0185	0.473	0.376–0.808
	SE (spring; ref.)	−0.069	0.128	0.291	0.5891	0.933	0.625–2.207
	PA	−0.480	0.214	3.895	0.0517	0.618	0.512–1.724
	Y	−0.028	0.007	11.049	0.0398	0.972	0.952–0.991

b, regression coefficient; SE, standard error; CI, confidence interval; ref, reference. S, sex; LS, litter size; KBW, kids birth weight; DMY, daily milk yield; SE, kidding season; PA, parity order; and Y, kidding year.

## Data Availability

Data are confidential, and their availability returns to the authors’ permission.
